# Rrd1 isomerizes RNA polymerase II in response to rapamycin

**DOI:** 10.1186/1471-2199-11-92

**Published:** 2010-12-03

**Authors:** Nathalie Jouvet, Jeremie Poschmann, Julie Douville, Lisa Bulet, Dindial Ramotar

**Affiliations:** 1 Maisonneuve-Rosemont Hospital, Research Center, Department of Immunology and Oncology, University of Montreal, 5415 de l'Assomption, Montreal, Quebec, Canada, H1T 2M4; 2 Genome Institute Singapore, 60 Biopolis Street, #02-01, Genome, Singapore 138672

## Abstract

**Background:**

In *Saccharomyces cerevisiae*, the immunosuppressant rapamycin engenders a profound modification in the transcriptional profile leading to growth arrest. Mutants devoid of Rrd1, a protein possessing *in vitro *peptidyl prolyl *cis/trans *isomerase activity, display striking resistance to the drug, although how Rrd1 activity is linked to the biological responses has not been elucidated.

**Results:**

We now provide evidence that Rrd1 is associated with the chromatin and it interacts with RNA polymerase II. Circular dichroism revealed that Rrd1 mediates structural changes onto the C-terminal domain (CTD) of the large subunit of RNA polymerase II (Rpb1) in response to rapamycin, although this appears to be independent of the overall phosphorylation status of the CTD. *In vitro *experiments, showed that recombinant Rrd1 directly isomerizes purified GST-CTD and that it releases RNA polymerase II from the chromatin. Consistent with this, we demonstrated that Rrd1 is required to alter RNA polymerase II occupancy on rapamycin responsive genes.

**Conclusion:**

We propose as a mechanism, that upon rapamycin exposure Rrd1 isomerizes Rpb1 to promote its dissociation from the chromatin in order to modulate transcription.

## Background

Rapamycin is an immunosuppressant that was recently approved for treating kidney carcinomas [1]. It is known to inhibit the Tor1 (Target of Rapamycin) kinase signalling pathway leading to growth inhibition [2]. In *S. cerevisiae*, several factors have been identified through genome-wide screens that when deleted cause resistance to rapamycin [3]. One of these proteins is Rrd1 (Rapamycin Resistance Deletion 1) that was first reported to play a role in protecting cells against oxidative DNA damage caused by the carcinogen 4-nitroquinoline-1-oxide (4-NQO) and by UVA [4]. Mutants deficient in Rrd1 are also unable to undergo rapamycin-induced growth arrest and therefore exhibit marked resistance to the drug [5]. Rrd1 is conserved in eukaryotes and shares 35% identity with the human phosphotyrosyl phosphatase activator, hPTPA, which was initially isolated as a protein that stimulated the weak phosphotyrosyl phosphatase activity of the type 2A Ser/Thr phosphatase PP2A [6,7]. We and others reported that Rrd1 can physically interact with the Ser/Thr phosphatase Sit4, a PP2A like phosphatase [8-10]. In *S. cerevisiae*, rapamycin binds to the peptidyl-prolyl *cis/trans *isomerase Fpr1 and this drug-protein complex inactivates the Tor1 kinase causing a profound modification in the transcriptional profile, and culminating in G1 growth arrest [11-13]. Inhibition of Tor1 leads to the activation of Sit4, by virtue of its dissociation from the inhibitor complex Tap42-Sit4, which in turn dephosphorylates several targets including the nutrient-responsive transcriptional activator Gln3 that translocates to the nucleus to activate *GLN1 *and *MEP2 *expression [13-15]. However, these Sit4-dependent processes do not require the function of Rrd1, suggesting that the latter protein might execute a function downstream in the Tor1 signalling pathway [12,16-18].

Recent data indicate that Rrd1 exerts an effect at the transcriptional level [12,16-18]. Genes known to be upregulated (e.g., the diauxic shift genes *CPA2 *and *PYC1*) and down-regulated (e.g., the ribosomal protein genes including *RPS26A*, *RPL30*, and *RPL9*) following rapamycin exposure showed an altered transcription pattern in *rrd1*Δ mutants [12,16-18]. To date, the exact function executed by Rrd1 causing alteration in transcription has not been investigated. Rrd1 and its mammalian counterpart PTPA have been shown to possess an *in vitro *peptidyl prolyl *cis*/*trans *isomerase (PPIase) activity on model substrates [19]. PPIases are ubiquitous proteins that catalytically facilitate the *cis/trans *isomerization of peptide bonds N-terminal to proline residues within polypeptide chains [20,21]. Both Rrd1 and PTPA can independently change the structure of short peptides including the synthetic substrate (^186^LQEPHEGPMCDL^198^) representing a conserved sequence amongst PP2A phosphatases [19]. As such, it has been suggested that Rrd1/PTPA could activate PP2As *via *this PPIase activity [19]. So far, neither the *in vivo *target nor the biological function of the PPIase activity of Rrd1 has been elucidated, although this is not the case for other PPIases. For example, the PPIases Ess1 and Pin1 from *S. cerevisiae *and mammalian cells, respectively, possess the ability to associate with the C-terminal domain (CTD) of Rpb1 [22,23]. In yeast, the CTD consists of 26 repeats of the YS_2_PTS_5_PS_7 _heptad sequence and Ess1 has been shown to stimulate the dephosphorylation of Ser-5 to efficiently terminate transcription of a subset of genes [24].

In this study, we show that Rrd1 is associated with RNA pol II and isomerizes the CTD of Rpb1 *in vivo *and *in vitro*. Our data suggest a model whereby this isomerization leads to the dissociation of RNA pol II from the chromatin resulting in transcriptional changes. This study provides insight into a possible new mechanism by which RNA pol II could rapidly respond to transcriptional changes.

## Methods

### Strains, media and plasmids

The strains used in this study were the parents BY4741 (*Mat a*, *his3-1*, *leu2-0*, *met15-0*, *ura3-0*), YDL401 (*MAT*a *his3*Δ*200 leu2*Δ*1 trp1 ura3-52 gal2 galΔ108*), and the isogenic mutants *rrd1*Δ and *gln3*Δ. Strains were endogenously and independently tag at the following loci *APN1*, *RAD52*, *RRD1*, *SWE1 *and *YAP8 *as previously described [25]. Strains bearing Rpb1-TAP was provided by Tom Begley (Albany, USA). Strains were grown in either rich (YPD) or selective (SD) media. Construction of pGFP-SIT4, pGFP-RAD52, pGFP-RRD1, GST-APN1 was previously described [8]. pGST-CTD was constructed by amplifying the murine CTD from plasmid pGCTD [26] and subcloned into pTW340 (provided by Tom Wilson, Michigan, USA). Construction of the plasmid pGAL-HIS-RRD1 and purification of HIS-Rrd1 fusion protein were done as previously reported for pHIS-BLH1 [27].

### Spot test analysis

The assay was done as previously described, except that plates contained rapamycin [28].

### Extraction of chromatin-associated proteins

Extraction of proteins bound to chromatin was done as previously described, except for the high salt extraction [29].

### Co-Immunoprecipitation experiments

Co-immunoprecipitation was done as previously described [8], except using 8WG16 antibody (Covance) covalently coupled to AminoLink matrix (Pierce) and total extracts [30] prepared from cells expressing either MYC- or GFP-tagged form of the indicated proteins or from the untagged parent or *rrd1*Δ mutant cells. The matrix with bound proteins was washed four times with a buffer containing 50 mM Tris-HCl (pH 7.5), 150 mM NaCl, and 0.1% NP40. The input (5%) used in the co-immunoprecipitation experiment as well as half the volume of the matrix were assessed by Western blot using either anti-MYC, -GFP (Clontech), or -ubiquitin (Rockland). The remaining half of the matrix was analyzed separately by Western blot probed with 8WG16 antibody.

### GST and GST-CTD purification

Strains bearing either pGST (this laboratory) or pGST-CTD plasmid were subcultured in 500 ml selective media to an OD_600 _of ~1.0, then treated with the appropriate drug for the indicated time. Cells were centrifuged, washed once with sterile water, and resuspended in 1.5 ml of yeast extraction buffer and extracts were prepared as above. The extracts were centrifuged at 3000 rpm in an Eppendorf centrifuge at 4°C for 3 min. Lysates were diluted 3-4 folds in PBS and Triton X-100 was added to a final concentration of 0.2%. One and half ml of glutathione sepharose 4B matrix (Pharmacia) was equilibrated with 50 ml of PBS in 50 ml Falcon tube then the lysate (~80 mg) was added and allowed to bind for one hour at room temperature on a rotating platform. The matrix was washed 3 times with PBS then transferred to 10 ml disposable column (BioRad). Excess of PBS was allowed to flow through, then GST-CTD was eluted with 10 fractions each of 150 μl of 50 mM Tris-HCl pH 9.0, 20 mM reduced glutathione (Sigma). Peak fractions were pooled to a total volume of 750 μl and the buffer was exchanged to 500 μl phosphate buffer using centricon (Millipore). Purity of the samples was verified by SDS-PAGE followed by silver staining.

### Purification of Rpb1-TAP

Proteins were extracted from untreated or rapamycin-treated (200 ng/ml for 1 h) cells as above and 2 mg were added to 40 μl of pre-equilibrated calmodulin affinity beads (Stratagene, USA). Purifications and washes were performed as described for the batch purification protocol provided by the manufacturer (Stratagene, USA). Eluates (50 μl) were collected, boiled and loaded onto SDS-PAGE for Western analysis. After probing with H5 or H14 antibody (Covance) membranes were stripped and re-probed with anti-PAP antibody (Sigma, USA).

### Western blot analysis of GST, GST-CTD and Rpb1-TAP

BY4741 parent or *rrd1*Δ mutant cells expressing the GST-CTD or carrying the endogenous Rpb1-TAP tag were subcultured in the appropriate media and treated with rapamycin (200 ng/ml for 30 min). Whole cell extracts or where indicated affinity purified proteins (GST, GST-CTD or Rpb1-TAP using manufacturer's protocol (Stratagene, USA)) were analyzed by Western blot with anti-GST (Sigma), H5 (anti-Ser2 phosphorylated) and H14 (anti-Ser5 phosphorylated) antibodies (Covance) or anti-PAP (Sigma).

### Interaction between Rrd1-MYC and GST-CTD

Total protein extracts derived from parent cells (100 ml) expressing GST-CTD or GST-Apn1, untreated or treated with rapamycin (200 ng/ml for 2 h) were allowed to bind to 1 ml GST affinity matrix slurry as described for the purification, except samples were not eluted from the columns. A second protein extract (1 mg) derived from a strain expressing Rrd1-MYC or Yap8-MYC was applied and allowed to bind for 1 h at room temperature on a rotating platform. The columns were then washed with 20 bed volumes of PBS and an aliquot of the beads (30 μl) was loaded onto an 8% SDS-PAGE and processed for Western blot. The presence of GST-CTD on both columns was detected using polyclonal anti-GST (Sigma) and the bound Rrd1-MYC was revealed using anti-MYC monoclonal antibody (SantaCruz).

### Circular dichroism spectroscopy

Continuous far-UV circular dichroism spectra (197-250 nm) of the GST and the GST-CTD fusion protein (2.0 μg and 4.32 μg, respectively, in 100 μl of 10 mM phosphate buffer pH 7.0, 50 mM NaCl) were collected using a Jasco-810 spectropolarimeter. The measurements were carried out at room temperature using a 1 mm path-length cuvette (Hellma) and a 1 nm bandwidth. Three spectra were collected for each sample and averaged. The spectral contribution of the buffer was corrected for by subtraction. Relative ellipticity was converted to mean residue molar ellipticity [Θ] according to Fasman [31].

### Limited chymotrypsin digestion assay

The purified GST-CTD (~100 ng) derived from parent cells untreated or treated with rapamycin (200 ng/ml for 2 h) was subjected to digestion with 5 ng chymotrypsin (Roche) in the presence of 1 mM CaCl_2_, and incubated at 37°C for the indicated time. Digestion was stopped by the addition of SDS-PAGE loading buffer and boiling of the samples. Processing of the GST-CTD was analyzed using 8% SDS-PAGE followed by staining with silver.

### *In vitro *isomerase assay

Purified HIS-Rrd1 (from *E. coli *using Talon affinity column according to the manufacturer (GE) protocol) was added to the purified GST-CTD in sodium phosphate buffer (10 mM NaPO_4 _pH 7.0, 50 mM NaCl) without or with 1 mM MgCl_2_, and 1 mM ATP in a final volume of 200 μl. The proteins were incubated for 1 h at 30°C the GST-CTD was recovered by GST-affinity purification and then subjected to CD analysis.

### *In Vitro *Rpb1 release assay

Exponentially growing culture (200 ml) of the BY4741 *rrd1*Δ Apn1-MYC strain was prepared and lysed as above for the extraction of chromatin associated proteins. Supernatant was discarded and the pellet was washed once in 1 ml of isomerization buffer (10 mM NaPO_4 _pH 7.0, 50 mM NaCl, 1 mM MgCl_2_, and 1 mM ATP). Supernatant was discarded again and pellet was resuspended in 600 μl of isomerization buffer and equally divided in three tubes. Increasing amounts of purified HIS-Rrd1 were added and samples were rocked for 1 h at 30°C. Samples were then spun down and supernatant was kept for subsequent western blot analysis. The remaining pellet was resuspended in benzonase buffer (50 mM Tris pH 8.0, 1 mM MgCl_2_) and 1 μl of benzonase (Novagen) was added and tubes were incubated for 30 min at 37°C. Supernatant (SOL) and chromatin (CHR) fractions were loaded onto SDS-PAGE gels for Western blot analysis with 4H8 (Cell Signaling) and anti-MYC antibodies.

### ChIP assay

The ChIP assay was done as previously described [32]. Primers are available upon request. *ACT1 *was used as an endogenous control and relative quantity was calculated using the ΔΔCT method (Applied Biosystems). IP's were normalized to the respective input. Untreated IP samples were given an arbitrary unit 1 and increase or decrease folds were calculated. At least three independent experiments were done for each gene and Student T test was used to calculate the p-value.

## Results

### Rrd1 is associated with the chromatin and interacts with Rpb1

We previously demonstrated that Rrd1 is required to modulate the expression of a subset of rapamycin-regulated genes independently of Sit4 [18]. To corroborate our earlier findings that Rrd1 acts separately from the Sit4-Gln3 signaling pathway, we deleted the *RRD1 *gene in the *gln3*Δ background (known also to be resistant to rapamycin) and examined the resulting *gln3Δ rrd1*Δ double mutant for the level of resistance to the drug [33]. This genetic analysis revealed that the *gln3Δ rrd1*Δ double mutant was significantly more resistant to rapamycin than either of the single mutants (Additional file 1 Figure S1), suggesting that Rrd1 performs a distinct role to regulate response to the drug.

**Figure 1:**
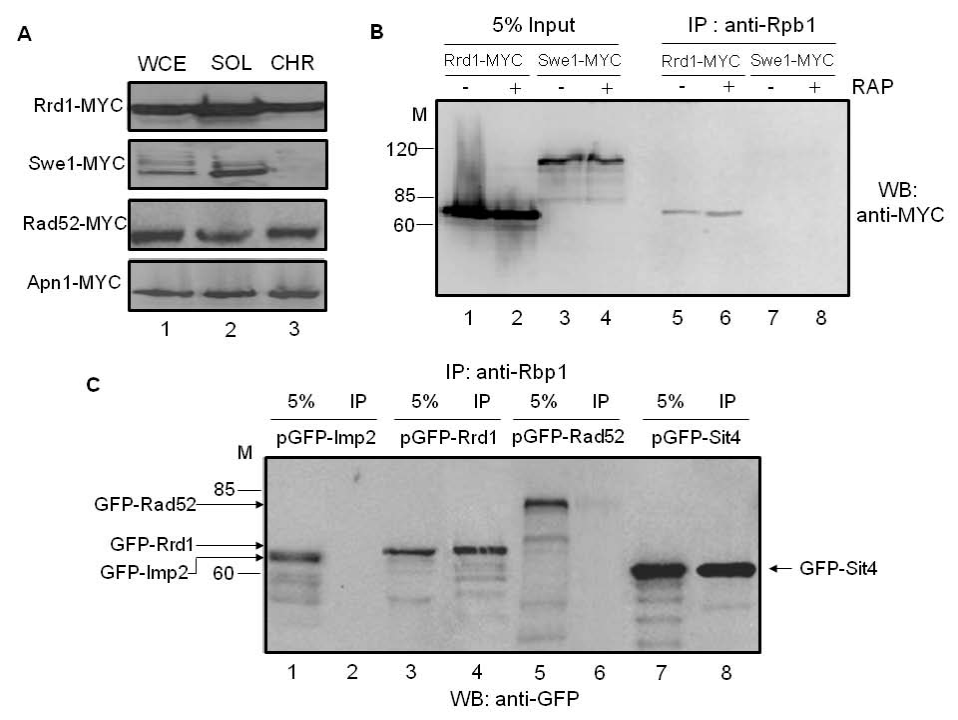
**Rrd1 is associated with the chromatin and interacts with Rpb1**. **A) **Rrd1 is bound to chromatin. Whole cell extract (WCE), soluble (SOL) and chromatin (CHR) fractions were derived (see Methods) from the parent cells expressing either Rrd1-MYC, Swe1-MYC, Rad52-MYC or Apn1-MYC and the distribution of the MYC-tagged proteins was examined by Western blots. The data is representative of two independent analyses. **B) **Rpb1 pull-down of Rrd1. The 8WG16 antibodies were used to immunoprecipitate extracts from untreated (-) and rapamycin-treated (+) (200 ng/ml for 30 min) cells expressing either Rrd1-MYC or Swe1-MYC. The presence of Rrd1 in the immunoprecipitates was determined by Western blotting. **C) **Specificity of Rpb1 pull-down of GFP tagged proteins. The 8WG16 antibodies were used to immunoprecipitate extracts from cells expressing either of the following GFP tagged proteins: Imp2, Rrd1, Rad52 or Sit4. The presence of the GFP-tagged proteins in the immunoprecipitates was detected by Western blotting using GFP antibody.

To investigate this potentially novel role of Rrd1, we first checked whether Rrd1 binds to chromatin in light of its involvement in gene regulation [18]. Chromatin fractions were derived from strains expressing MYC-tagged Rrd1, as well as the control proteins Swe1, Rad52 and Apn1 from the endogenous loci and subjected to Western blot analysis probed with anti-MYC antibody. As shown in Figure 1A, a significant amount of Rrd1-MYC was found in the chromatin fraction (lane 3), suggesting that Rrd1 is associated with the chromatin and consistent with an earlier study showing that Rrd1 is also present in the nucleus [8]. In contrast, the control protein Swe1-MYC was only found in the soluble fraction (lane 2), while Rad52-MYC and Apn1-MYC, two DNA repair proteins known to bind chromatin, were present in the chromatin fraction (lane 3) [34,35].

Since Rrd1 is bound to the chromatin and is involved in regulating gene expression, we tested if it is associated with RNA pol II by performing co-immunoprecipitation analysis. For this experiment, we used total extracts derived from cells expressing either Rrd1-MYC or Swe1-MYC and checked for the pull-down with anti-Rpb1 (8WG16). Rrd1-MYC was co-immunoprecipitated with Rpb1, but not the control protein Swe1-MYC (Figure 1B). Since only a small amount of Rrd1-MYC was co-immunoprecipitated with anti-Rpb1, the association between Rrd1 and RNA pol II may be weak or transient. There was no alteration in the amount of Rrd1 co-immunoprecipitated by anti-Rpb1 when cells were treated with rapamycin (200 ng/ml for 30 min) (Figure 1B).

Anti-Rpb1 also co-immunoprecipitated Rrd1 from parent cells carrying a plasmid expressing GFP-tagged Rrd1 (Figure 1C). In addition, the Sit4 phosphatase known to physically interact with Rrd1 [8] co-immunoprecipitated with Rpb1 from parent cells expressing this protein as GFP fusion (Figure 1C). Two additional GFP fusion proteins, GFP-Imp2 and GFP-Rad52, which do not interact with Rrd1, were not co-immunoprecipitated with anti-Rpb1 antibody, although a minute amount of GFP-Rad52 non-specifically interacted with the beads used for immunoprecipitation (Figure 1C, and data not shown). Thus, Rpb1 associates with proteins known to bind Rrd1, suggesting that Rrd1 could exist in a complex with Rpb1. We note that the reverse co-immunoprecipitation with Rrd1-MYC did not pull down Rpb1 under the same reaction conditions, raising the possibility that the size of the RNA pol II complex might impede the pull down although we cannot exclude other alternatives such as a weak or indirect interaction via another protein.

### Rrd1 associates with the CTD of Rpb1 and alters its structure in response to rapamycin

**Figure 2:**
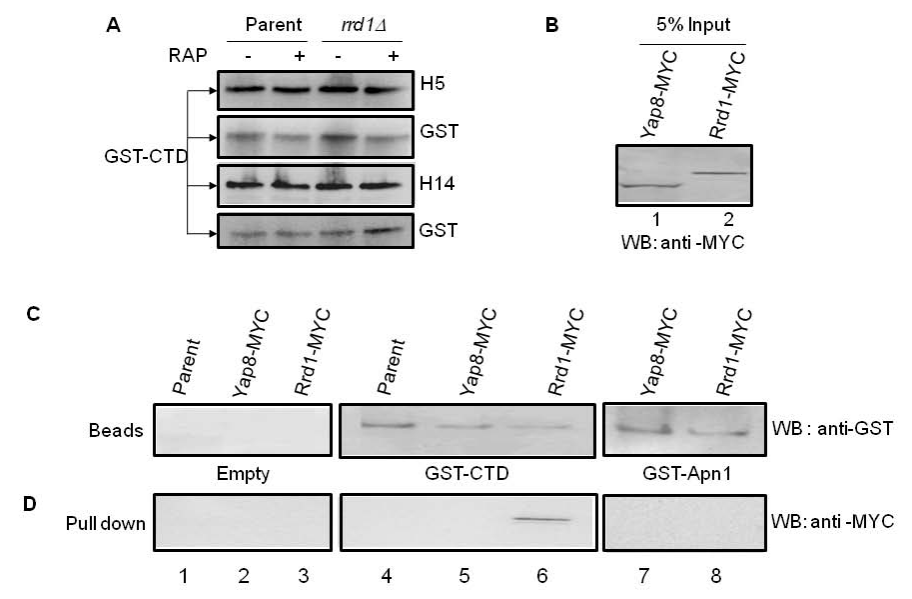
**Analysis of the GST-CTD and its interaction with Rrd1**. **A) **Comparison of the expression and phosphorylation status of the GST-CTD between parent and *rrd1*Δ mutant cells following rapamycin exposure. The indicated cells expressing GST-CTD were treated with (+) and without (-) rapamycin (200 ng/ml for 30 min) and total protein extracts were probed for Ser-2 phosphorylation (H5) or Ser-5 phosphorylation (H14). The membranes were stripped and reprobed with anti-GST antibody. **B-D) **Retention of Rrd1-MYC by GST-CTD affinity beads. **B) **The input (5% of the total amount of protein extracts added to the beads) of parent cells expressing Yap8-MYC and Rrd1-MYC from the endogenous locus. Western blot analysis was done using anti-MYC antibody. **C) **and **D) **Total protein extracts derived from the parent or parent expressing either Yap8-MYC or Rrd1-MYC were incubated with the empty beads or beads containing either GST-CTD or GST-Apn1 (see Methods). The beads were then washed and an aliquot examined for retention of the MYC tagged proteins using anti-GST antibodies (C) or anti-GST antibodies (D). Results shown are representative of two independent experiments.

Since the C-terminal domain (CTD) of Rpb1 is a repeated sequence (YSPTSPS) rich in proline residues, and has previously been shown to bind the isomerases Ess1 and Pin1 [22,23,36], we reasoned that Rrd1 could function to isomerize the CTD. As such, we assessed whether the CTD is a substrate for the PPIase activity of Rrd1 *in vivo*. The CTD was expressed as a GST fusion protein from a previously described plasmid (see Methods) and has been shown to undergo post-translational modifications including Ser-5 and Ser-2 phosphorylation, isomerization and ubiquitylation [24,26,37,38]. Introduction of this plasmid into the parent and *rrd1*Δ strains directed the expression of the GST-CTD fusion protein with the expected size (95-kDa) as determined by Western blot analysis probed with anti-GST antibodies (Figure 2A, see also Additional file 1 Figure S2). The GST-CTD contained both phosphorylated Ser-5 and Ser-2 as detected by anti-H14 and anti-H5 antibodies, which specifically recognize Ser-5 and Ser-2 phosphorylation, respectively (Figure 2A), consistent with previous studies that the GST-CTD can be functionally modified *in vivo *[26,37-39]. From these analyses, we observed no differences in the (i) size, (ii) level of expression, and (iii) phosphorylation of the GST-CTD whether it was derived from the parent or the *rrd1*Δ mutant or from cells that were pretreated with rapamycin (Figure 2A Additional file 1 Figure S2).

We next prepared GST-CTD affinity beads from parent cells and determined whether these could pull down Rrd1. Total extract derived from the parent strain expressing Rrd1-MYC (Figure 2B, lane 2) was incubated with the GST-CTD affinity beads. The beads were recovered, washed and an aliquot examined for retention of Rrd1-MYC by Western blot analysis. As shown in Figure 2C and 2D, Rrd1-MYC was pulled down by the GST-CTD affinity beads. In contrast, the GST-CTD affinity beads did not pull down the transcriptional activator Yap8, also tagged with MYC (Figure 2B, C and 2D). As expected, the empty beads did not pull down Rrd1-MYC from the total extract nor did the control beads carrying GST-Apn1 (Figure 2C and 2D). These data support the notion that Rrd1 associates with the CTD of Rpb1, consistent with the above observation that Rpb1 co-immunoprecipitated Rrd1.

**Figure 3:**
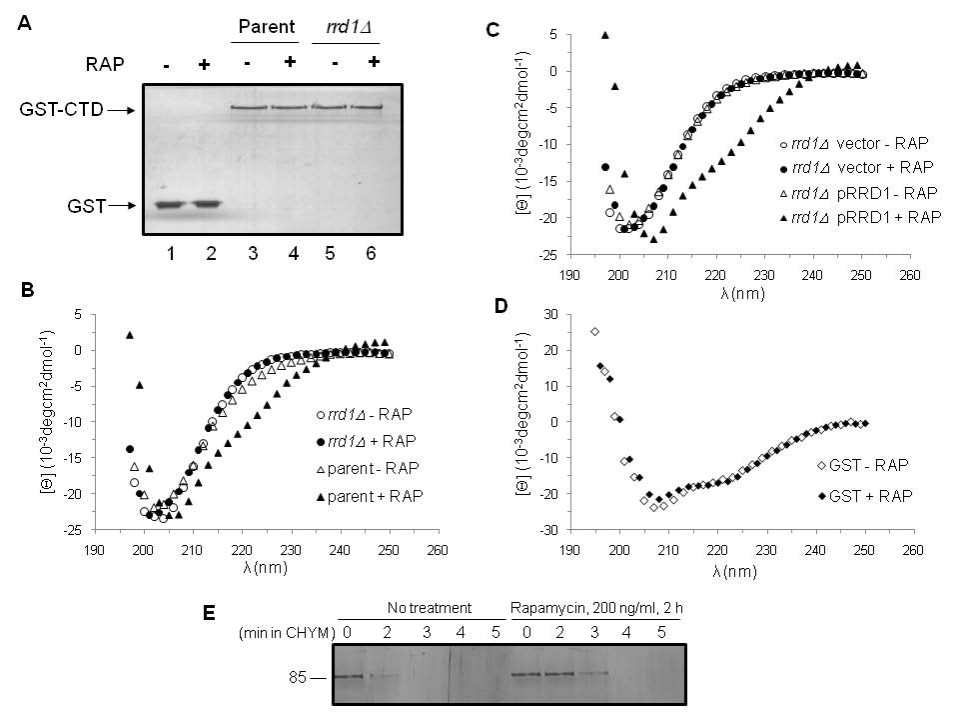
***rrd1*Δ mutants are unable to induce conformational changes to the GST-CTD in response to rapamycin**. **A) **Silver stained gel of purified GST and GST-CTD. The indicated strains carrying either the GST (lanes 1 and 2) or GST-CTD expressing plasmid (lanes 3-6) were untreated (-) or treated (+) with rapamycin (RAP) (200 ng/ml for 30 min). **B) **Far-UV circular dichroism (CD) spectral analysis of purified GST-CTD. The purified GST-CTD (0.45 μM) was derived from the parent strain (triangle) or *rrd1*Δ mutant (circle) that were untreated (opened symbol) or treated (closed symbol) with rapamycin. **C) **Far-UV CD spectral analysis of purified GST-CTD. The purified GST-CTD (0.45 μM) was derived from the *rrd1*Δ mutant carrying the empty vector (circle) or the pRRD1 plasmid (triangle) that were untreated (opened symbol) or treated (closed symbol) with rapamycin. **D) **CD analysis of purified GST (0.76 μM) derived from untreated (opened symbol) and rapamycin treated (closed symbol) parent cells as above. Results shown are the averages of two independent experiments. **E) **Limited proteolysis of purified GST-CTD derived from parent cells untreated or treated with rapamycin. The purified GST-CTD was subjected to partial chymotrypsin digestion and analyzed by silver staining. Results shown are representative of two independent experiments.

We next investigated whether Rrd1 could induce conformational changes in the GST-CTD fusion protein by using circular dichroism (CD) spectroscopy, a method that is very sensitive to changes in the secondary structure of proteins [31]. We first purified the GST-CTD from the parent and the *rrd1*Δ mutant, as well as GST from the parent to be used as the control. Silver stain analysis of the purified GST-CTD revealed that there was no difference in the size of this protein, whether it was derived from the parent or the *rrd1*Δ mutant (Figure 3A, lane 3 vs. 5) or when the cells were treated with rapamycin (lane 3 vs. 4 or 5 vs. 6). As observed for total extract, the purified GST-CTD also contained both phosphorylated forms, Ser-2 and Ser-5, but showed no alteration in response to rapamycin (Additional file 1 Figure S2A). To ensure that the observed phosphorylation status of the GST-CTD is similar to Rpb1 CTD phosphorylation, we purified Rpb1 from the TAP tagged strains and monitored this protein for its phosphorylation. Like the GST-CTD, Rpb1-TAP showed no differences in either Ser-5 or Ser-2 phosphorylation following rapamycin treatment (Additional file 1 Figure S2B). However, this approach may not distinguish between subtle phosphorylation differences that may occur amongst the heptad repeats [40]. Since the GST-CTD is similarly phosphorylated as the endogenous Rpb1, we used it as a tool for further analysis.

CD spectra obtained for the purified GST-CTD derived from either the untreated parent or *rrd1*Δ mutant were indistinguishable, and displayed a minimum at 202 nm (Figure 3B). In contrast, GST-CTD derived from the parent cells treated with rapamycin exhibited a spectrum with a minimum at 208 nm and shoulder at ~225 nm (Figure 3B), suggesting that the GST-CTD underwent a detectable change in its secondary structure. Remarkably, rapamycin treatment of the *rrd1*Δ mutant failed to induce this conformational change onto the GST-CTD (Figure 3B). Introduction of a single copy plasmid expressing functional Rrd1 in the *rrd1*Δ mutant restored the change in the spectral pattern of the GST-CTD (Figure 3C) [4]. Additionally, purified GST alone derived from untreated or rapamycin treated parent cells did not exhibit any structural differences, suggesting that it is the CTD portion of the fusion protein that is undergoing the rapamycin-induced changes (Figure 3D). We further confirmed the structural change of the GST-CTD as observed by CD using limited proteolysis with chymotrypsin, which can distinguish proteins with different secondary structures and exclusively cleaves peptides in the trans-proline conformation [41]. As shown in Figure 3E, the GST-CTD purified from the rapamycin-treated parent cells was more resistant to limited chymotrypsin digestion, as opposed to the GST-CTD derived from the untreated cells, suggesting that indeed the GST-CTD went through a structural reorganization in response to rapamycin. On the basis of these findings, it would appear that the CTD of Rpb1 changes its structure *in vivo *following exposure to rapamycin, and that Rrd1 is essential for this alteration.

### Rrd1 alters the GST-CTD structure in response to 4-NQO, but not MMS

**Figure 4:**
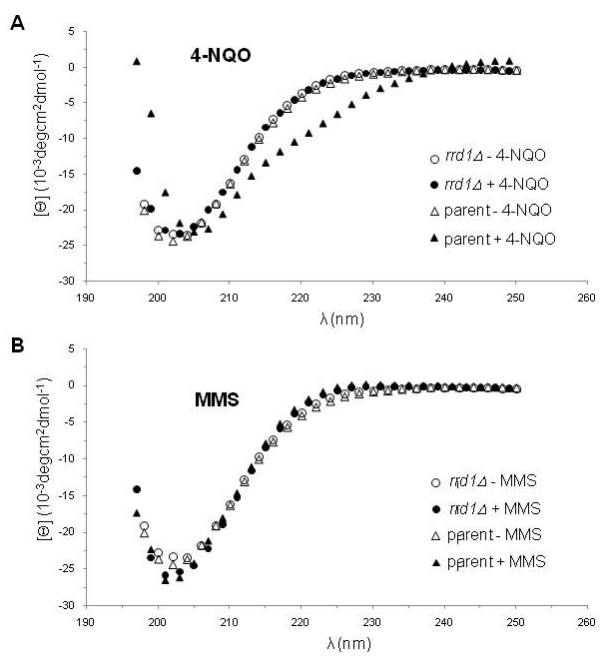
**4-NQO, but not MMS, induces structural changes onto the GST-CTD**. **A and B) **CD analysis of the purified GST-CTD derived from exponentially growing parent (triangle) and *rrd1*Δ mutant (circle) that were untreated (opened symbol) or treated (closed symbol) with either 4-NQO (2 μg/ml 30 min) panel A or MMS (1% for 60 min) panel B.

We next checked if isomerization of the CTD is specific for rapamycin. Since the *rrd1*Δ mutant was previously shown to be sensitive to the DNA damaging agent 4-NQO [4], which induces oxidative stress as well as creating bulky lesions onto the DNA [42], we examined for isomerization of the GST- CTD in the parent and the mutant following treatment with this drug. We observed that the structure of the GST-CTD was altered in the parent, but not in the *rrd1*Δ mutant following 4-NQO treatment (Figure 4A). We also tested another DNA damaging agent, methyl methane sulfonate (MMS) (Figure 4B), to which the *rrd1*Δ mutant displays parental sensitivity [4]. MMS creates apurinic/apyrimidinic sites in the genome, and for this experiment it was used at a concentration that kills ~70% of the cells. Under this condition, the GST-CTD showed no structural alteration following the MMS treatment (Figure 4B). On the basis of these findings, it would appear that this phenomenon might occur for other stress conditions besides exposure to rapamycin.

### Rrd1 directly alters the structure of the CTD in vitro

**Figure 5:**
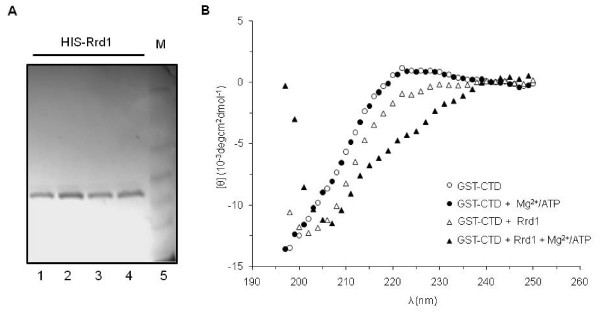
**Purified recombinant Rrd1 alters the structure of purified GST-CTD *in vitro***. **A) **Silver stained gel of purified recombinant HIS-Rrd1 from *E. coli *expression system (see Methods). Lanes 1-2 and 3-4 are elution samples from two independent purifications obtained directly from Talon affinity column; lane 5, molecular weight standard. **B) **Equimolar amounts (4.5 μM) of purified GST-CTD derived from the *rrd1*Δ mutant and the purified recombinant HIS-Rrd1 (triangle) were incubated at 30°C in phosphate buffer in the absence (opened symbol) and presence (closed symbol) of Mg^2+^/ATP. The resulting GST-CTD was re-purified free of the recombinant HIS-Rrd1 and subjected to CD analysis as in Figure 3. The result is the average of two independent experiments.

We next examined whether purified Rrd1 can induce structural changes onto the CTD *in vitro*. To do this, we incubated equimolar amounts of recombinant HIS-Rrd1 purified from *E. coli *(Figure 5A) with affinity purified GST-CTD derived from the *rrd1*Δ mutant at 30°C for 30 min, and then recovered the GST-CTD for CD analysis. As shown in Figure 5B, purified HIS-Rrd1 significantly modified the CTD structure under the standard phosphate buffer reaction conditions. Since the Rrd1 isomerase activity has been shown to be stimulated by ATP and Mg^2+ ^[19], we examined the effect of these additions to the reaction mixture. Inclusion of ATP and Mg^2+ ^in the buffer caused no structural alteration to the CTD in the absence of Rrd1 (Figure 5B). However, addition of purified HIS-Rrd1 to the complete ATP/Mg^2+ ^phosphate buffer introduced a more dramatic change to the CTD structure, as compared to the mixture lacking ATP/Mg^2+ ^(Figure 5B). Moreover, the purified HIS-Rrd1 did not confer any structural changes onto another purified GST fusion protein, GST-Apn1 (data not shown). These findings suggest that Rrd1 can directly isomerize the CTD.

### Comparison of RNA pol II occupancy at rapamycin-responsive genes

**Figure 6:**
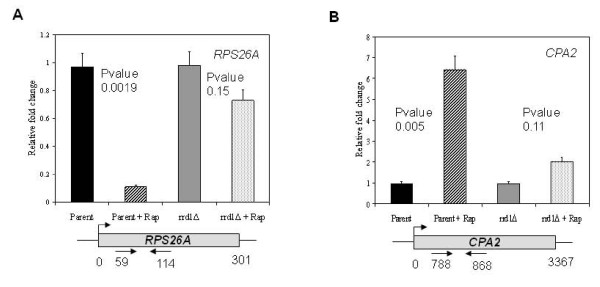
**Comparison of RNA pol II occupancy at the indicated target genes in the parent and *rrd1*Δ mutant strain in response to rapamycin treatment**. Cells were untreated or treated with 200 ng/ml rapamycin for 30 min and Rpb1 localization was analyzed by ChIP assay (see Methods). Primer locations are indicated below the diagram. The respective input normalized IP amounts were quantified relative to the *ACT1 *gene using the ΔΔCT method. Results are shown as the average of three independent experiments. Error bars represent standard deviation and the *P*-values compare untreated vs. treated.

Since Rrd1 associates with and isomerizes the CTD, and that *rrd1*Δ mutant did not affect the phosphorylation status of Rpb1, we asked whether it would alter RNA pol II occupancy on rapamycin responsive genes *in vivo*. To do this, we performed chromatin immunoprecipitation (ChIP) analysis of Rpb1 on two known RNA pol II-responsive genes, *RPS26A *and *CPA2 *[32]. Since both genes are known to be rapidly downregulated and upregulated, respectively, within 30 min, we treated cells for this time period with rapamycin [11,18]. In parent cells, the Rpb1-ChIP signal from the *RPS26A *gene was reduced by nearly 8-fold upon rapamycin treatment (Figure 6A). In contrast, Rpb1 remained associated with *RPS26A *in the *rrd1*Δ mutant (Figure 6A). In the case of the upregulated gene *CPA2*, we observed an increase in Rpb1-ChIP signal in the parent upon rapamycin, whereas in the mutant there was only a modest increase in the signal (Figure 6B). The occupancy of RNA pol II on these genes is consistent with the mRNA expression levels [11,18]. These data raise the possibility that Rrd1 might displace Rpb1 in order to optimize rapid transcriptional changes caused by rapamycin.

### Purified Rrd1 stimulates the release of chromatin-bound RNA pol II in vitro

**Figure 7:**
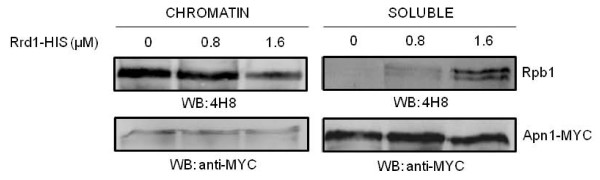
**Purified recombinant Rrd1 dissociates Rpb1 from the chromatin *in vitro***. Increasing amounts of purified HIS-Rrd1 were added to the chromatin fraction isolated from *rrd1*Δ mutant strain expressing Apn1-MYC and incubated at 30°C for 1 h in phosphate buffer. Chromatin was recovered from the buffer and both fractions were analyzed by Western blotting probed with 4H8 (against Rpb1) and anti-MYC antibodies. Apn1-MYC was used as loading control. Result shown is representative of at least three experiments.

To explore the above possibility, we examined if purified Rrd1 would displace RNA pol II from the chromatin. Briefly, we isolated chromatin containing RNA pol II derived from the *rrd1*Δ mutant, the chromatin was washed and resuspended in the standard phosphate buffer containing ATP and Mg^2+^. To this reaction, increasing amounts of purified Rrd1 was added and following incubation the levels of chromatin-bound and soluble Rpb1 were monitored by Western blot. As shown in Figure 7 increasing concentration of Rrd1 caused a loss of chromatin-bound Rpb1, while there was a correlating gain in the soluble fraction. In contrast, Rrd1 concentration did not affect the level of the control protein Apn1-MYC. Collectively, our data indicate that Rrd1 possesses the ability to isomerize the CTD of Rpb1 thereby promoting its displacement from the chromatin.

## Discussion

In the present study, we show that Rrd1 is a chromatin bound protein, which associates with RNA pol II and presumably through the CTD of Rpb1. We believe that this association allows isomerization of the CTD in response to specific stress such as that caused by rapamycin and 4-NQO. In addition, we show that *in vitro *purified Rrd1 (i) can directly alter the structure of the CTD and (ii) dissociate Rpb1 from the chromatin. On the basis of these observations, we propose the following model whereby in response to specific stress conditions the RNA pol II associated Rrd1 isomerizes the CTD of Rpb1 such that the polymerase is dissociated from the chromatin. Once the RNA pol II is released it would be recruited to stress-responsive genes.

There is supporting evidence that elongating RNA pol II is in excess on ribosomal protein genes, surprisingly associated with a low transcriptional rate under glucose grown conditions [43]. However, once these cells are submitted to a metabolic change, e.g., a switch to galactose growth conditions, the level of RNA pol II decreased on these ribosomal genes and the transcriptional rate increased [43]. This shift also simultaneously caused an enrichment of RNA pol II onto mitochondrial genes [43]. This suggests a mechanism where excessive RNA pol II is removed from the ribosomal genes and recruited to mitochondrial genes to increase expression. Therefore, metabolic switches would stimulate re-localization of elongating RNA pol II from one regulon to the other. As it is known that rapamycin mimics starvation conditions and represses ribosomal biogenesis, we suspect a similar mechanism as the glucose-galactose shift is operational to rapidly change transcription. Besides Rrd1, another well characterized peptidyl prolyl isomerase Pin1 can trigger the release of RNA pol II from transcribing genes in human cells [23]. Under normal conditions, Pin1 interacts with the phosphorylated CTD of RNA pol II and this association is retained along the length of transcribed genes [23]. However, when Pin1 is overexpressed it promotes hyperphosphorylation of the CTD during the transition from initiation to elongation, thereby causing RNA pol II to dissociate from active genes and leading to the inhibition of transcription [23,44]. The dissociated RNA pol II accumulates in enlarged speckle-associated structures enriched for transcription and RNA processing factors [23,45].

Because Rrd1 intersects with the biological functions of Pin1, it is possible that Rrd1 could modulate the phosphorylation status of the CTD. Recent studies showed that the yeast homologue of Pin1, Ess1, binds and catalyzes the *cis/trans *isomerization of the CTD such that Ser-5 phosphorylation can be dephosphorylated by the Ssu72 phosphatase [24]. Moreover, a variant of Ess1 (Cys120Arg) caused accumulation of Ser-5 phosphorylation, and not Ser-2 phosphorylation, both of which were monitored using the same set of antibodies (anti-H5, -H14 and -8WG16) as in this study [24]. We found no alteration in the global Ser-2 and Ser-5 phosphorylation status upon rapamycin treatment, as well as between the parent and the *rrd1*Δ mutant using the same set of antibodies (Figure 2A and Additional file 1 Figure S2). As such, it would seem that Rrd1 uses a novel mechanism independent of phosphorylation to isomerize the CTD, although we cannot exclude the possibility that there are unique Ser-2 and Ser-5 phosphorylation differences which can be masked by neighboring phosphorylations, for example, where one heptad is phosphorylated, but not the adjacent [40]. However, since RNA pol II exists in different phosphorylation forms throughout the transcription cycle, it seems logical to have a mechanism that triggers RNA pol II release independent of its phosphorylation status.

In yeast, the CTD consists of 26 repeats of the heptad sequence YSPTSPS. It exists largely in a disordered structure, but adopts a static conformation upon interaction with target proteins such as the mediator complex that regulates transcription initiation and enzymes that modify the 5' and 3'ends of mRNA [46,47]. Binding of these proteins to the CTD is modulated by serine phosphorylation and proline isomerization [40]. Thus, a given heptad repeat could give rise to many different conformations with the various combinations of phosphorylated Ser-2, -5 and -7, as well as the *cis*/*trans *isomerization of the two prolines, Pro-3 and Pro-6, to generate a broad range of binding sites to allow precise association with several factors [46-48]. At least three CTD interacting proteins (Pcf1, Pin1, and Ctg-1 from *C. albicans*) have been shown to bind exclusively the all-*trans *conformation, providing support for the hypothesis that proline isomerization of the CTD plays a critical regulatory role [48]. This strongly suggests that multiple conformations of the CTD exist *in vivo*. Consistent with this notion, we observed by CD analysis two conformations of the CTD that remained stable throughout its purification (Lisa Miller, Brookhaven National Laboratories, personal communications) from untreated and rapamycin-treated cells (Figure 3). These different conformations could be the result of proline isomerization, as prolines are known to be stable in either the *cis *or *trans *conformation when the protein is in a folded form [49]. Only peptidyl prolyl isomerases such as Pin1/Ess1 are known to trigger a switch between the *cis *and *trans *conformations of the CTD [50], and that in the absence of these enzymes the conformational switch is slow [51]. Because Rrd1 possesses peptidyl prolyl isomerase activity and it associates with RNA pol II, it seems likely that this function is responsible for inducing structural changes to the CTD upon rapamycin exposure. In support of this, Rrd1 directly alters the CTD structure *in vitro *(Figure 5), and we therefore predict that Rrd1 might act in a similar manner onto the CTD *in vivo*.

In addition to rapamycin, we also observed that the DNA damaging agent 4-NQO, but not MMS, triggered alteration of the CTD structure (Figure 4). We examined the effect of 4-NQO, as we had previously shown that *rrd1*Δ mutants were sensitive to this agent and not to MMS [4]. The distinct difference between 4-NQO and MMS is that the former agent potently induces the production of reactive oxygen species such as superoxide anions [42]. Both starvation and oxidative stress are known to mediate similar transcriptional programs, also termed as the environmental stress response, for example, where ribosome biogenesis is turned off [51,52]. This would explain why the *rrd1*Δ mutants are sensitive to 4-NQO, but resistant to rapamycin; (i) genes required for counteracting the 4-NQO-induced oxidative stress are not turned on efficiently and as a result the cells accumulate genotoxic lesions, and (ii) under rapamycin condition nutrients are still available and the failure to alter gene expression allows *rrd1*Δ mutants to grow.

## Conclusions

Taken together, our data suggest that Rrd1 participates in a novel mechanism that allows redistribution of RNA pol II for transcriptional regulation of genes involved in specific stress conditions. These results provide the first direct evidence that Rrd1 acts *in vivo *as an isomerase and establish a physiological function for this activity.

## Authors' contributions

NJ carried out chromatin extraction, co-immunoprecipitation, Western blot analysis, protein purification and CD analysis, and drafted the manuscript. JP carried out Western blot analysis, genetic analysis, ChIP analysis, *in vitro *chromatin assay and drafted the manuscript. JD carried out chromatin extraction, co-immunoprecipitation, Western blot analysis, protein purification and CD analysis, and drafted the first version of the manuscript. LB carried out the GST-CTD and Rrd1-MYC pull-down. DR helped conceive the study, participated in its design and coordination, and assisted with writing the manuscript. All authors read and approved the final manuscript.
